# Treatment of thrombosis in KD Patients using tissue plasminogen activator: a single center study

**DOI:** 10.1186/s12969-022-00767-7

**Published:** 2022-12-05

**Authors:** Yanqiu Chu, Yunming Xu, Ce Wang, Xiaona Yu, Quanmei Ma, Hong Wang

**Affiliations:** 1 Pediatric department of shengjing hospital, China Medical University, 110004 shenyang, China; 2 Ultrasonic department of shengjing hospital, China Medical University, 110004 shenyang, China; 3 Radiology department of shengjing hospital, China Medical University, 110004 shenyang, China

**Keywords:** Children, Kawasaki disease, Coronary aneurysm, Thrombosis, Alteplase, Heparin, Warfarin, Prognosis

## Abstract

**Objective:**

The most severe complication associated with giant coronary aneurysm in children with Kawasaki disease is ischemic cardiomyopathy (ICM) caused by thrombosis. Addition of tissue plasminogen activator, Alteplase, in the treatment regimen can be an efficient thrombolytic therapy, and therefore can have a significantly positive impact on patients’ quality of life in long term.

**Methods:**

Total four male KD patients with central thromboses in coronary aneurysm were treated in Pediatric Cardiology Department of Shengjing Hospital, China Medical University, from January 2020 to August 2021. These patients received thrombolytic treatments including Alteplase once + Heparin for 1 week followed by continuous oral Warfarin + Aspirin + Clopidogrel.

**Results:**

4 young male KD patients had coronary aneurysm (CAA) complicated with total 7 occurrences of central thrombosis. These patients were given alteplase and heparin/oral Warfarin + Aspirin + Clopidogrel treatment. 9 days to 2 months later, thromboses were significantly dissolved. The treatment successfully diminished the thrombosis complication.

**Conclusion:**

1. Pediatric KD patients complicated with coronary aneurysm thrombosis are prone to recurrence of thrombosis. 2.  In KD patients complicated with coronary aneurysm thrombosis, treatments described in Method can be used for treating either small thromboses formed less than 1 month with strong echo and convex lumen or large thromboses with mixed strong and weak echo. With these treatments, coronary artery blood flow can be improved or completely recovered. 3. Clinical experiences at our center in treating these KD patients suggest that Alteplase can be considered in thrombolytic treatment beyond the limitation of less than 12 h of thrombosis occurrence.

**Supplementary Information:**

The online version contains supplementary material available at 10.1186/s12969-022-00767-7.

Pediatric Kawasaki disease has replaced rheumatic disease as the first risk causing acquired heart diseases in developed countries, even in China and Indian [1–3], and CAA has been found associated with the acquired heart diseases in KD patients. According to guidelines by AHA 2004 and Japanese KD of the 5th edition, CAA is defined as coronary artery ≥ 4 mm or Z score ≥ 5. In these KD patients, the most serious complication is the thrombosis in CAA, which can impact patients’ life qualities [4]. Giant CAA is difficult to recover completely. Invasive treatment is generally not introduced in young patients until patients turn in adulthood. Treating thromboses effectively and as early as possible will benefit KD patients in terms of maintaining normal cardiac functions and development. A successful thrombolytic treatment can also eliminate ICM complication in these young KD patients. However, implementing a successful thrombolytic treatment regimen has been very challenging for pediatricians.

In adult patients with ischemia, significant symptoms can develop in a short period of time, and the timing of thrombosis can be assessed clearly. Therefore, physicians can manage thrombolytic therapy including Alteplase in adult patients within less than 12 h of myocardial infarction onset, as recommended by instructions of drug usage. On the other hand, in pediatric KD patients, thromboses occur in dilated coronary aneurysms, and they develop slowly. It is unlikely to develop complete blood occlusion in a short period of time. Therefore, the clinical symptoms may not be as significant as in adult patients with ischemia [5]. In addition, it is difficult for very young kids to accurately describe specific symptoms associated with timing of ischemia. As a result, in young KD patients, thrombolytic therapy is rarely implemented, and clinical experiences are limited [4]. At present, Alteplase has been reported only being used in 2 infants with coronary artery thrombosis (one 29-day-old- and the other 3-month-old) in United States [6, 7]. One adult KD patient with coronary aneurysm thrombosis received a treatment with combination of alteplase and actilyse for thrombosis [8]. Currently, anticoagulation and antiplatelet are main choices used for treating thrombosis. Nevertheless, at our clinic center, the data collected from treatment experiences suggests that anticoagulation and antiplatelet treatments have better results in preventing thrombosis than in treating existing thromboses. Here, we present data to support a thrombolytic treatment regimen in pediatric KD patients with thrombosis using Alteplase beyond 12 h of thrombus onset.

## Methods

Total 4 KD boys with thrombosis in CAA and convex to lumen were treated in Shengjing Hospital affiliated with China Medical University from January 2020 to August 2021. Data including ECG, ECHO, and CTA were collected for analysis in terms of CAA location and thrombosis. Clinical symptoms, treatment methods, testing results and prognosis through the treatment were also followed and analyzed.

### Inclusion criteria

i) symptoms and signs in the acute phase met the diagnostic criteria of KD/IKD [4]; ii) confirmed coronary aneurysms or giant aneurysm in coronary arteries using ECHO or coronary CTA at any test time point, according to KD Diagnostic Criterion (2002) of the Kawasaki Disease Research Committee of the Ministry of Health and Welfare of Japan [9], and guidelines by the American Heart Association (AHA) for the diagnosis of Kawasaki disease and atypical Kawasaki disease in 2017. iii) Convex lumen thrombosis in coronary aneurysms confirmed by ECHO or CTCA.

### Exclusion criteria

1) No follow-up; 2) Without anticoagulant or thrombolytic therapy; 3) attached CAA wall without effects on blood flow.

### Treatment

#### Thrombolytic therapy

Alteplase, intravenous infusion at 1.5 mg/kg in 2 h [10].

#### Anticoagulant therapy

A: Heparin 100U/kg, once 8 h, intravenous injection (IV) [11]. B: Warfarin with the first dose of 0.2 mg/kg.d followed by oral 0.1 mg/kg.d on the next day. DIC was monitored and maintained INR between 2 and 3 [12];

#### Anti-platelet therapy

A: oral Aspirin at 3-5 mg/kg.d for QD. B: oral Clopidogrel bisulfate at 1 mg/kg.d for QD. C: Dipyridamole at 3-5 mg/kg.d, divided into BID.

#### Treatment regimen definition

- Method 1: warfarin + asprin + dipyridamole/clopidogrel to treat patients with giant CAA but without thrombosis.
- Method 2: heperin + asprin + dipyridamole/clopidogrel for 7–10 days followed by Method 1 to treat patients with giant CAA and small thromboses with low density.
- Method 3: Heperin 10U/kg iv, then Alteplase once + asprin + dipyridamole/clopidogrel, followed by Method 2 to treat patients with giant CAA + giant thromboses or small thromboses with high density.

### Statistic analysis

The outcomes of Method 1, 2, and 3 treatments were analyzed and compared using descriptive methods.

## Results

The 4 pediatric KD patients had total 7 occurrences of thrombosis detected as central thromboses in CAA. The average age of patients over the treatment period was 4 years and 5 months old (1.5-8 years old). They received total 5 Alteplase treatments. Brief patients’ medical histories, thrombosis locations, blood test results, and treatment descriptions are included in Table 1.

**Table 1:** The treatment in KD patients with thrombosis in CAA

Patient ID		Treatment at beginning				Thrombosis occurrence and treatment							ECG
		**Age** **(y)**	**Time-**_Asp_ _(d)_	**Time-**_IVIG_ _(d)_	**IVIG** **dose**	**Time from KD onset**	**WBC** (×10^9^)	**PLT** (×10^9^)	**CRP** (mg/L)	**Treatment Method**	**Duration to recover**	**Site**	
**1**	**1st**	**6.5**	**16**	**16**	**2 g/kg**	**16d**	**12.8**	**505**	**126.8**	**2**	**10d**	**LAD**	**Normal**
	**2nd**	**6.7**	**60**	**-**	**-**	**2 m**	**5.4**	**343**	**2.78**	**3**	**2 m**	**LAD**	**Normal**
**2**	**1st**	**6.5**	**9**	**9d/14d**	**4 g/kg**	**1 m**	**12.6**	**548**	**6.8**	**3**	**10d**	**Central RCA**	**Normal**
	**2nd**	**8**	**9**	**8 m**	**2 g/kg**	**1.5y**	**4.37**	**289**	**5.7**	**3**	**> 2 m**	**Distal RCA**	**Abnormal**
**3**	**1st**	**1.5**	**13**	**13**	**2 g/kg**	**5.5**	**6.12**	**277**	**-**	**2**	**-**	**LAD**	**Normal**
	**2nd**					**6.5y**	**4.31**	**302**	**1.9**	**3**	**8 m**	**LAD**	**Normal**
**4**		**3**	**15**	**16**	**2 g/kg**	**15d**	**5.59**	**521**	**39.7**	**3**	**10d**	**LM**	**Normal**

**Fig. 1:**
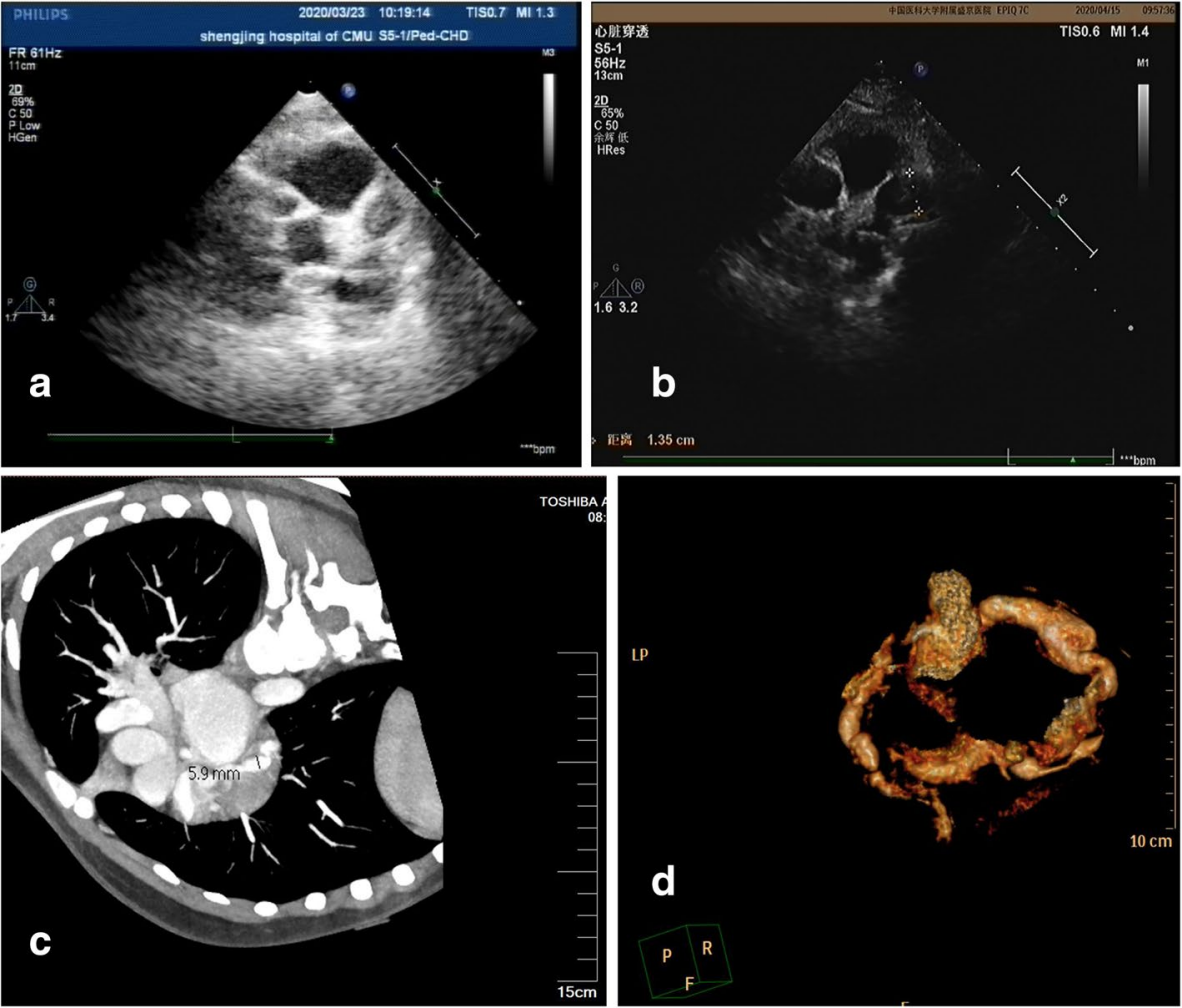
In Patient 1, ECHO showed the thrombus formed in the LAD and the lumen was nearly blocked (**a**) 2 months after the onset of the disease. After 2 months of thrombolysis treatment, the thrombus disappeared completely (**b**), and no contrast filling defect was found in the LAD in CTCA (**c**). Bilateral coronary arteries presented with irregular and marked dilation (**d**)

**Fig. 2:**
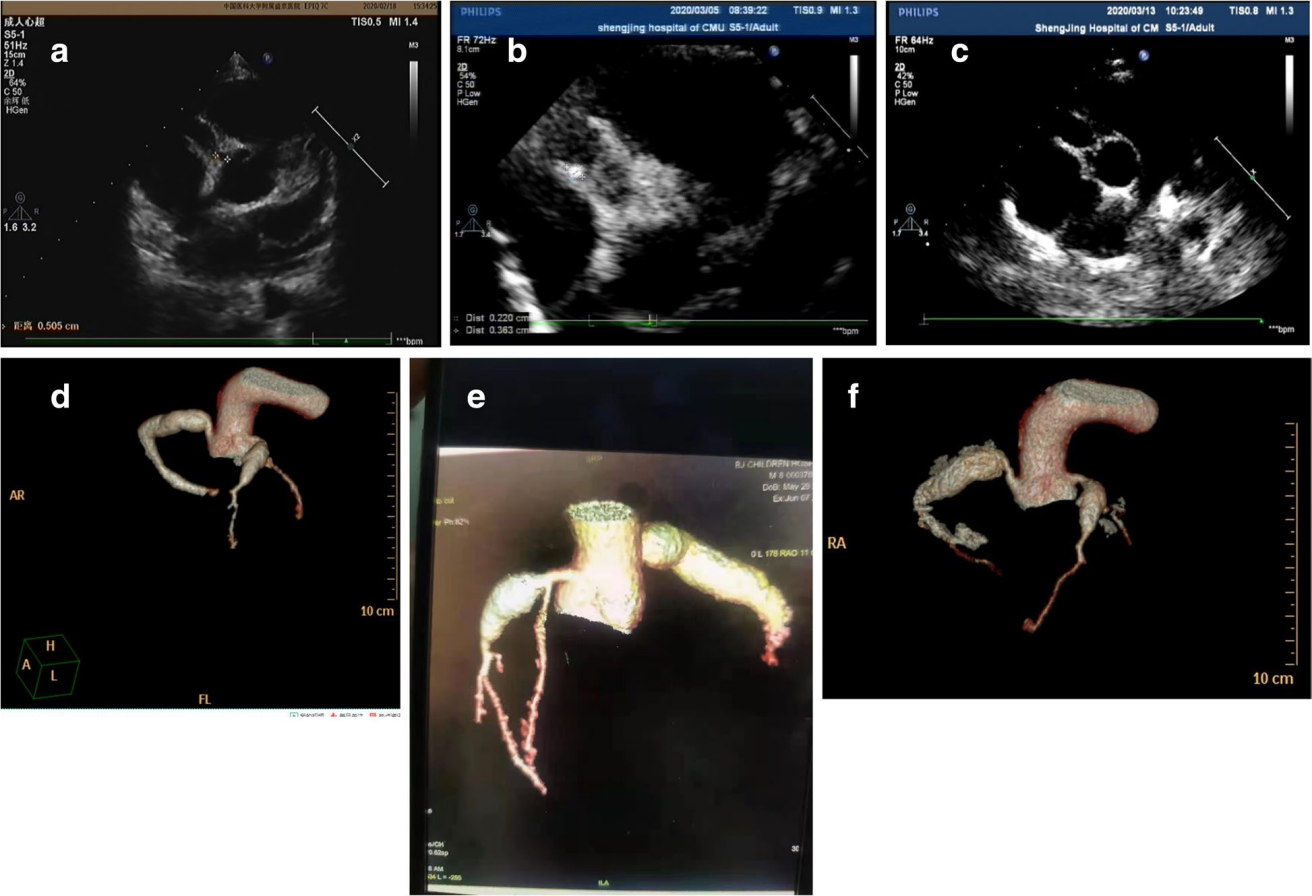
In Patient 2, ECHO showed RCA dilated to 9 mm at 16 days of illness (**a**). At 30 days of illness, thrombus with relatively high density (2.6 × 3.2 mm, b-red arrow) was found and convex to the lumen in RCA (**b**). The thrombolytic treatment began six days later, and the thrombus disappeared after 10 days treatment (**c**). Coronary artery CTCA showed aneurysm in RCA and distal blood flow were clearly at 30 days of illness (**d**). At 17 months of illness onset, CTCA at a different hospital detected reoccurred thrombosis at the distal end of RCA, and it blocked the blood flow (**e**). After thrombolysis treatment for 2 months, the distal blood flow almost recovered (**f**)

**Fig. 3:**
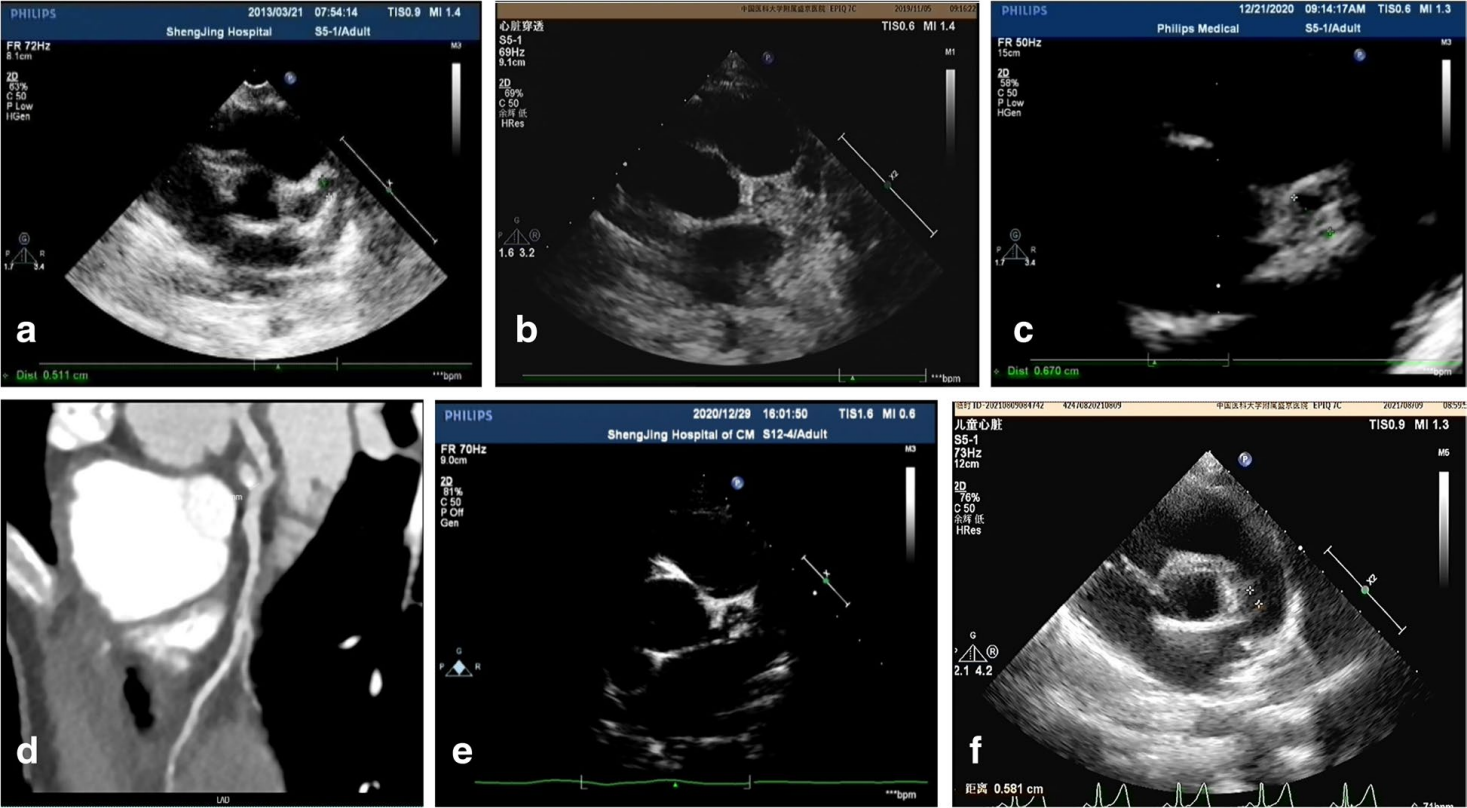
ECHO showed LAD aneurysmal dilatation at 14 days of illness in Patient 3 (**a**). At 5.5 years of illness, ECHO showed strong density thromboses in LAD (**b**). At 6.5 years of illness, ECHO showed mixed density thromboses (the strong echo mixed with the weak echo) in LAD (**c**) CTCA showed LAD dilatation with contrast agent filling defect (thrombus) (**d**). After 9 days of thrombolytic/anticoagulant/antiplatelet treatment, the low density portion (the newly formed thrombus) disappeared and patient’s symptoms of chest tightness disappeared (**e**). After 8 months of thrombolytic/anticoagulant/antiplatelet treatment, the strong density portion (the previously formed thrombus) disappeared, too (**f**)

**Fig. 4:**
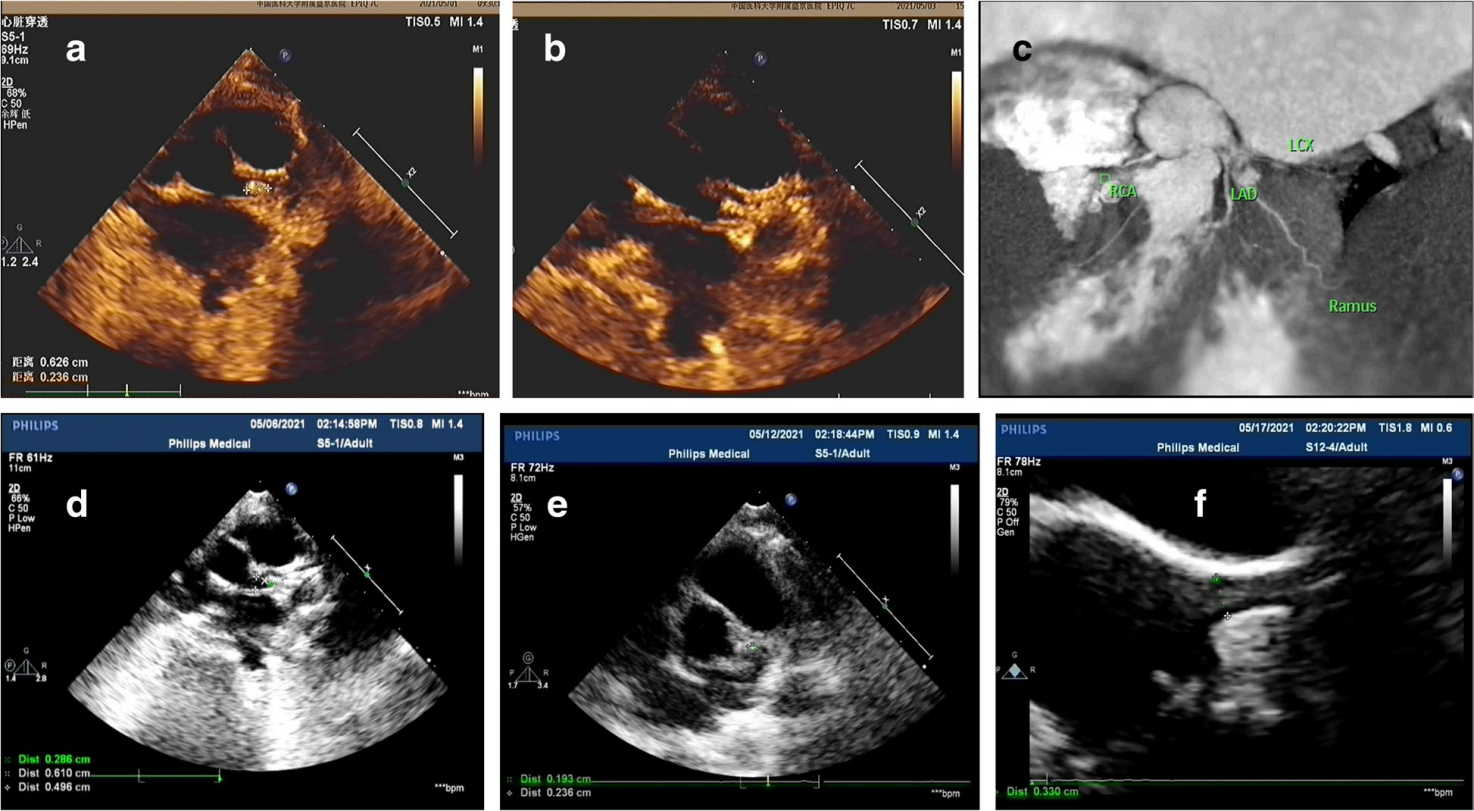
ECHO showed LM aneurysmal dilatation and filling with strong echo thrombosis 6.2 × 2.6 mm at 15 days of illness in Patient 4 (**a**). Treatment started with alteplase. 3days later, ECHO still showed thrombus in LM (**b**). At 22 days of illness onset, contrast filling defect wasn’t observed in CTCA (**c**) and at the same time ECHO showed a small thrombus in LM (**d**). At 27 days of illness, ECHO showed that the thrombus was reduced to 2.3 × 1.9 mm (**e**). At 32 days of illness, the thrombus was diminished in ECHO (**f**)

**Fig. 5:**
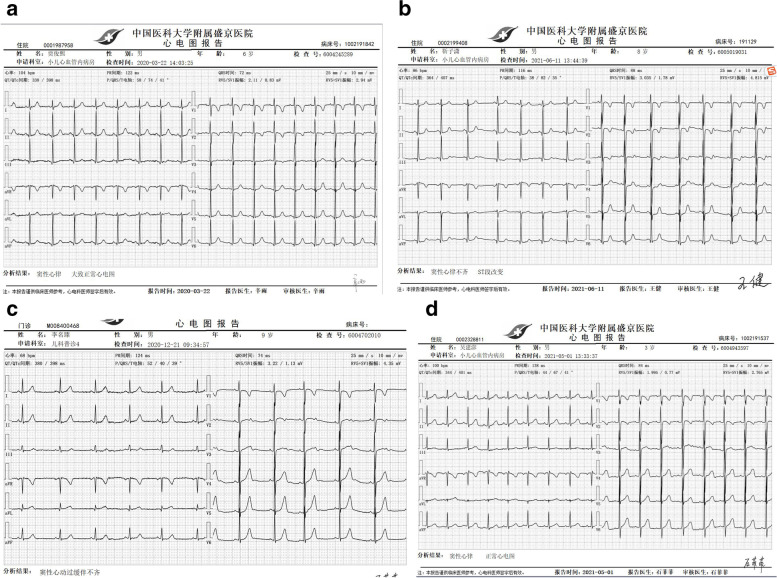
In patient 1, ECG was normal when thrombosis reoccurred in LAD at 2 months of KD onset (**a**). In Patient 2, ECG showed downward ST segment at the low and lateral wall when the thrombosis reoccurred at the distal end of RCA. The thrombosis blocked the blood flow at 16 months of KD onset (**b**). Patient 3 had a normal ECG results when he had chest pain during exercises, and the thrombosis was detected in LAD at 6.5 years of KD onset (**c**).  In Patient 4, ECG was normal when thrombosis was detected at 15 days from onset of illness (**d**)

For Patient #1, at the time when he was diagnosed with KD, a CAA was identified and a low-density thrombus was detected in the anterior descending (LAD). After 10 days of treatment with Method 2 (anticoagulant/antiplatelet), the thrombus disappeared (Table 1). However, at 2 months of illness diagnosis, ECHO detected reoccurred thrombosis with mixed echo in LAD (Fig. 1a). After treatment with Method 3 (thrombolytic/ anticoagulant/antiplatelet) for 2 months, reoccurred thromboses diminished (Fig. 1b, c and d). Patient #2 had developed thrombosis twice in RCA. The first high-density one was formed in the central RCA, in a small size (Fig. 2a and b). It was dissolved after Method 3 treatment for 9 days (Fig. 2c and d). Two years later, the second one was detected in the RCA and it blocked blood flow at distal RCA (Fig. 2e). After Method 3 treatment for 2 months, the recurred thrombosis was dissolved and the blood flow was recovered (Fig. 2f). Patient # 3 was diagnosed with KD when he was 1.5 years old and received treatment for CAA (Table 1). The patient stopped taking medicines for 4 years. 5.5 years after KD onset, he was found to have high-density thrombosis in LAD, but he did not have symptoms. He received Method 2 (heparin treatment followed by Method 1) for 10 days. However, there were no changes in thrombosis in the follow up test (Fig. 3a). 6.5 years after KD illness onset, the patient had chest pain after vigorous exercises. ECHO revealed a strong echo mixed with a weak echo, suggesting thromboses with mixed densities (Fig. 3b, c and d). The patient received Method 3 treatment for 9 days, and his symptoms of chest tightness were resolved. Echo test showed that the low-density portion of thromboses disappeared (Fig. 3e). Eight months later, the high-density thrombus was dissolved completely (Fig. 3f). In Patient # 4, ECHO showed LM aneurysmal dilatation filled with strong echo thrombosis 6.2 × 2.6 mm at 15 days of illness (Fig. 4a). The patient received Method 3 treatment. Three days after the treatment, the thrombosis was still present in LM (Fig. 4b). Seven days after the treatment, CTCA did not detect contrast filling defect (Fig. 4c), and ECHO showed the unchanged thrombosis in LM (Fig. 4d). Twelve days after the treatment, the size of thrombus was reduced to 2.3 × 1.9 mm, as shown in ECHO (Fig. 4e). Seventeen days after Method 3 treatment, ECHO revealed the thrombus was dissolved (Fig. 4f). The ECG changes in these four children at the time of thrombus discovery are attached (Fig. 5).

## Discussion

Cardiovascular manifestations and complications are the major contributors to morbidity and mortality associated with KD, both during the acute illness phase and in the long-term progression [9]. Rupture of CAA in KD is rare. If the blood flow in CAA is unobstructed, the cardiac function will not be significantly impacted. In the subacute stage and recovery stage of KD, the elevated platelet count and platelet activity will increase the risk of thrombosis [13]. Meanwhile, KD children with CAA usually do not respond to IVIG treatment very well, and therefore they will need repeated treatment with IVIG and glucocorticoids. Both drugs are know to increase platelet abnormally, increasing the risk of thrombosis [14–16].

Here we summarize the treatments with or without Alteplase for KD patients associated with thrombosis at our center over several years. In 2010 and 2015, we admitted two children (control case 1 and control case 2) with delayed diagnosis who developed LAD thrombosis and dilated cardiomyopathy. They were treated with Method 1, a combination of warfarin + aspirin + dipyridamole. At the end, the patient admitted in 2010 died in 2012. The patient admitted in 2015 is disable now [17] (Supplementary file: Figures 6 and 7). In current study, Patient 1 was admitted with confirmed the LAD thrombosis, similar to the two patients admitted in 2010 and 2015, and the blood flow was almost completely occluded when thrombosis reoccurred. After the thrombolytic treatment with Alteplase, then heparin for a week, followed by combination of warfarin + aspirin + clopidogre treatment, the thrombus diminished completely 2 months later in Patient (1) In 2018, a patient ( control case 3) with distal RCA thrombosis was treated with Method 2 (heparin for 1 week, followed by combination of warfarin + aspirin + dipyridamole). There were no changes in thrombosis when the patient was followed up 10 months later. CMR confirmed the formation of myocardial transmural necrosis in the inferior left ventricular wall, and ECG detected ST-T changes (Supplementary file: Fig. 8). It took 26 months for the thrombus partially dissolved. But the myocardial necrosis was irreversible. Patient 2 in current study had similar distal thrombosis in the RCA that disrupted blood flow. We approached with a thrombolytic treatment including Alteplase, then heparin for 1 week, followed by warfarin + aspirin + clopidogre treatment. After 2 months of treatment blood flow in the distal trunk of the RCA was significantly recovered. For patients who have low density thrombosis, a good prognosis can be achieved after receiving a treatment with heparin IV for 7 days followed by warfarin plus antiplatelet therapy (case 1 at the first occurrence of thrombosis) [18]. Patient 1 received this treatment of Method (2) When thromboses are large with mixed densities, projecting lumen or disrupting blood flow, these coronary thrombosis in KD children can be the indication of alteplase. In these patients, Alteplase was used in treating the thrombosis reoccurred in Patient 1, the thrombosis occurred and reoccurred in Patient 2, the mixed-density thrombosis occurred at different times in Patient 3, and the thrombosis occurred in Patient 4. Although the course of treatment is longer, the prognosis is good as long as the treatment persists.

Considering the slow formation of thromboses and restriction to vigorous activities by parents, most pediatric patients may develop adaption and do not present significant myocardial ischemia symptoms [19]. In KD Patient 2, the first coronary thrombosis did not occur at the peak of platelets but was detected in the descending phase of platelets 1 month after KD onset. The second thrombus formed when the platelets were in normal range, indicating a subacute stage of increased platelet activity when thrombosis was likely formed [3, 20, 21]. Aspirin and dipyridamole, besides their anti-platelet activities, can reduce tissue factor procoagulant activity. Aspirin inhibits the upstream of STING pathway [22]. In Patient 3, warfarin, asprin, and dipyridamole were used to treat bilateral coronary aneurysms. However, the treatment was discontinued due to bleeding. Hyperechoic thrombosis in the LAD was detected 5.5 years after the onset of the disease (over a year after the last ECHO examination). Treatment with Method 2 (haprin, warfarin, asprin, and dipyridamole) for half of a month failed to dissolve or minimize the thrombus). 6.5 years after the onset of the disease, The patient complained chest pain after strenuous activities. Tests revealed that thromboses with mixed densities were developed in LAD and almost blocked blood flow (possible myocardial ischemia). After treatment with Method 3, the thrombus with low density diminished 2 weeks later, and the patient resumed his daily activities. The high-density thrombosis dissolved 8 months later.

There are other choices for treating coronary thrombosis, such as interventional procedures and urokinase. Interventional procedures for coronary thrombolysis have been established for adult patients. However, the same procedures are not preferred in infant or young patients due to the much narrower coronary artery diameters [23–25]. In the United States, it has been reported that the first treatment for adult KD patients with coronary thrombosis is to perform thrombolysis in the coronary artery. If this first choice of treatment fails, intravenous thrombolysis should be continued, which achieves good thrombolysis results. At this time, it is far longer than 12 h, and the drug has been given for several times [17]. In Japan, it has been reported that intracoronary urokinase and post-thrombolytic regimen are successful in treating an infant patient with KD and acute myocardial infarction [26]. In our center, we used similar regimen to treat a 19-month-old KD patient who developed low-density thrombus within middle RCA. ECHO showed a poor Doppler color flow filling. CTCA showed uneven fillings at the proximal middle section of the RCA. The patient received treatment including urokinase, 40 000 U + 5%GC50ml, pumped at 50ml/h, once a day for 3 days, then heparin for 2 days, followed by Method 2 for 10 days. As a result, the low-density thrombus dissolved (Supplementary file: Fig. 9). This may suggest that, in KD patients who develop low density thromboses, urokinase with subsequent warfarin can be used to treat thrombosis successfully.

From January 2020 to May 2022, total 552 KD cases were diagnosed and treated in our center, including 540 new cases. Among them there were 23 cases with giant CAA, and 16 of them were from the new cases (2.96%). In 23 giant CAA cases, 8 developed coronary thrombosis (a small number of mural thrombosis was not counted). In current study, four pediatric KD patients received systemic thrombolytic therapy with alteplase in total 5 times, including the second thrombosis at LAD that almost blocked blood flow in Patient 1, the first high density thrombosis (3.6 mm x 2.2 mm) and the second thrombosis at the distal end of RCA that blocked blood flow in Patient 2, the old and new thromboses that almost blocked bleed flow at LAD in Patient 3, and thrombosis at LM bifurcation During Alteplase treatment, there were 1 case of bleeding from the gingival cavity and 1 case of bleeding from the skin puncture. In addition, one 19 months old boy with KD developed low density thrombus at inner right CAA. After a treatment including Urokinase, the low-density thrombus disappeared after 10 days. During the Urokinase treatment, there was no bleeding.

The side effects of alteplase in children mainly include bleeding in pursuance of pinhole and bleeding in the gingival area of caries, which usually occur within 1 h of medication. Because heparin can lead to thrombocytopenia, blood routine should be monitored during heparin administration [19]. Since coronary atherosclerosis in adults can be accompanied by cerebral atherosclerosis and gastrointestinal ulcers, the biggest risk by thrombolysis in treating these patients is internal bleeding. Thrombolytic therapy in pulmonary embolism is reported to likely cause bleeding [27]. However, in multivariate analysis, thrombolytic therapy using alteplase in patients with myocardial infarction/cerebral infarction has not been associated with gastrointestinal tract or cerebral hemorrhage [28]. Cerebral atherosclerosis and gastrointestinal ulcers are very rare in children. In young patients, bleeding usually occurs in superficial area where it will be easily observed (e.g. epistaxis, the site of skin puncture, caries oozing blood and so on). Bleeding can be stopped by local compression using cotton balls and reduction of the Alteplase infusion speed by half. Therefore, the risk is relatively low when using thrombolytic therapy in pediatric patients.

In addition, thromboses in adult patients with coronary heart diseases occur at inner coronary atherosclerosis stenosis lumen. The symptoms caused by thrombus blocking blood flow are obvious and can be described by patients, which helps capture onset time clearly. In children with KD, thrombosis occurs within dilated aneurysm, and the thrombus expands relatively slowly and rarely obstructs blood flow abruptly. It is also hard for young children to describe symptoms at the early stage. Therefore, it is challenging to manage the treatment within 12 h of onset in young patients. The beneficial effects of anticoagulant and antiplatelet drugs alone are limited. Intravenous alteplase is an excellent remedy, even more than 12 h after the onset of symptoms.

In conclusion, our clinical experiences in treating KD complicated with thrombus in CAA are summarized in the following. (1) Pediatric KD patients complicated with coronary aneurysm thrombosis are prone to recurrence of thrombosis. (2) When Giant CAA mural thrombus does not affect blood flow, anticoagulant and antiplatelet therapies are efficient enough. (3) For central thrombus, as long as DIC and platelets are within the safe range, it is not necessary to strictly limit the Alteplase treatment within < 12 h of thrombus occurrence.

## Supplementary Information

Additional file 1.

## Data Availability

The datasets used and analysis during the current study are available from the corresponding author on reasonable request.

## Abbreviations

ICM: Ischemic cardiomyopathy
KD: Kawasaki disease
IKD: Incomplete KD
CAA: Coronary artery aneurysm
ECG: Electrocardiograph
ECHO: Echocardiograph
CTCA: Computer tomography of coronary artery angiography
QD: Once a day
BID: Bis in die
LM: Left main coronary artery
LAD: Left anterior descending branch
LCX: Left circumflex artery
RCA: Right coronary artery
DIC: Disseminated intravascular coagulation
CMR: Cardiac magnetic resonance
LVED: Left ventricular end distolic diameter
LVEF: Left ventricular ejection fraction
