# Supplementary material for: Treatment of thrombosis in KD Patients using tissue plasminogen activator: a single center study
Source: Pediatr Rheumatol Online J. 2022 Dec 5;20:111. doi: 10.1186/s12969-022-00767-7 (PMC9720999; doi:10.1186/s12969-022-00767-7)
Supplement: Supplementary file 1 — Additional file 1. [file 12969_2022_767_MOESM1_ESM.docx]

**Figure 6. (Control case 1) He was 3 years old boy, ECHO showed LM aneurysmal dilation and with thrombus in it at 6 months later of misdiagnosis in case 1 (a-orange arrow). He was treated withe Warfarin, Aspirin, and Dipyridamole, LM was still aneurysmal dilation and with no thrombus in it at 10 months later of misdiagnosis (b), but LVED increased to 64mm (c). At 1 year later of misdiagnosis, LVEF decreased to 24%(d). CTCA showed LM dilatation and there was the filling defect near the LAD (e-****orange arrow)，the blood flow was almost interrupted in the LAD (f-black arrow). About 3 years later of misdiagnosis, he was sudden death.**


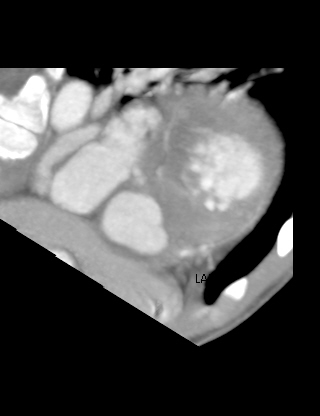


LAD

occlusion?


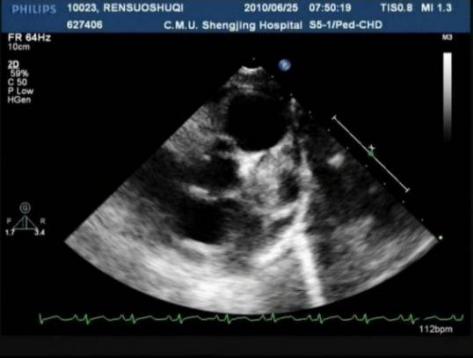

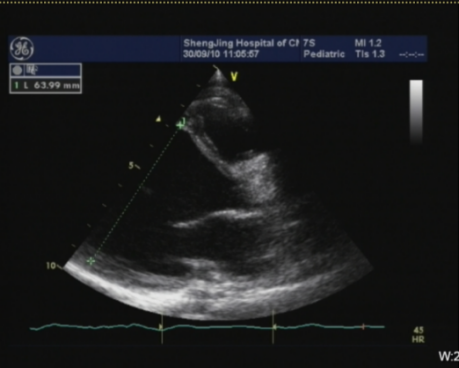

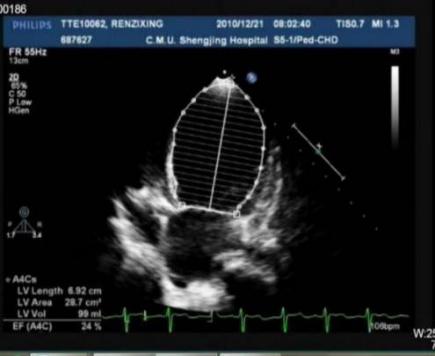

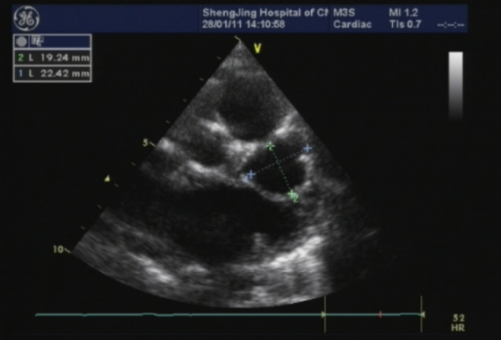

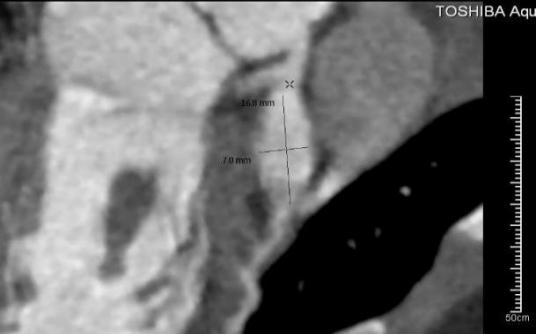


a

f

e

d

c

b

**Figure 7. (Control case 2) He was 3 years old boy, ECHO showed LM aneurysmal dilation and with thrombus in it at 6 months later of misdiagnosis in case 2 (a-orange arrow)，the blood flow almost cut off (b), LVEF 0.39 (c). He was treated withe Warfarin, Aspirin, and Dipyridamole. At 5.5 years of illness, the thrombus almost absorbed (d). LVED enlarged to 72mm (e).** **CMR showed transmural myocardial necrosis of the left ventricular free wall (f).**


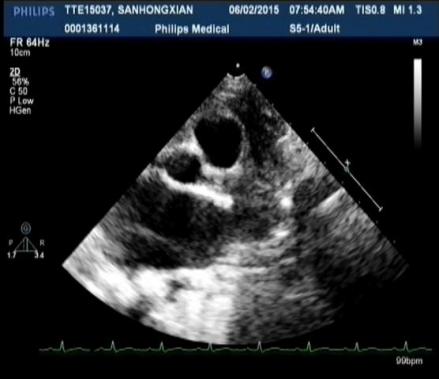

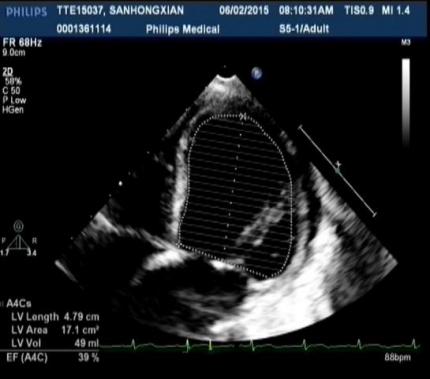

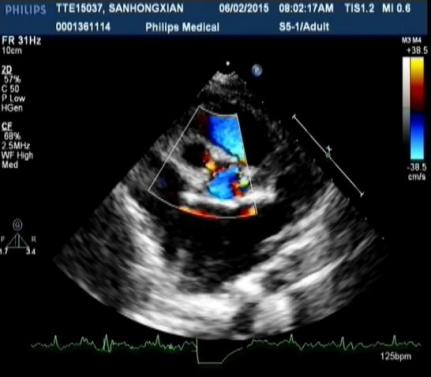

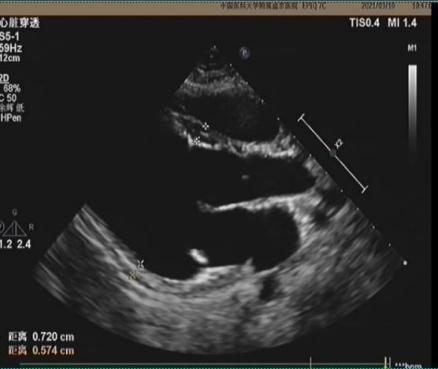

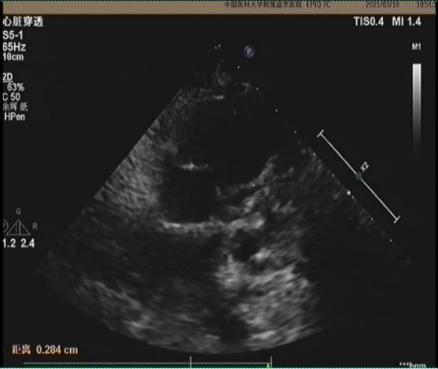


**a**

**d**

**e**

**c**

**b**


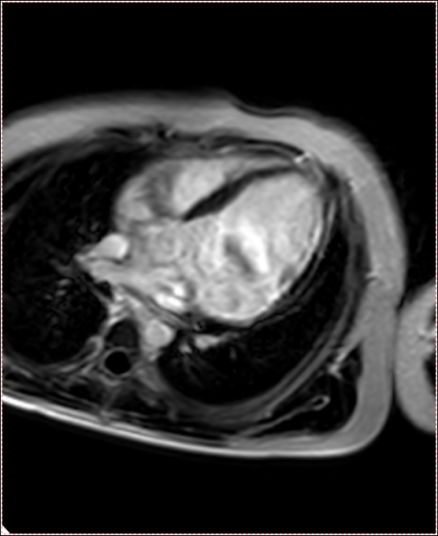


**d**

Figure 8. Imaging and ECG results for the patient admitted in 2018 (control case 3). CTCA showed RCA with mild dilatation and blood flow (a) at 20 days of illness. Six months later, ECG revealed the inverted or bidirectional T waves in the lower and lateral walls of the heart (b), CTCA confirmed distal RCA thrombosis that occluded blood flow (c). The patient was treated with Method 2. At 1.5 years of illness, CTCA showed the distal RCA thrombosis that occluded blood flow (d), and CMR indicated left ventricular transmural necrosis (e).


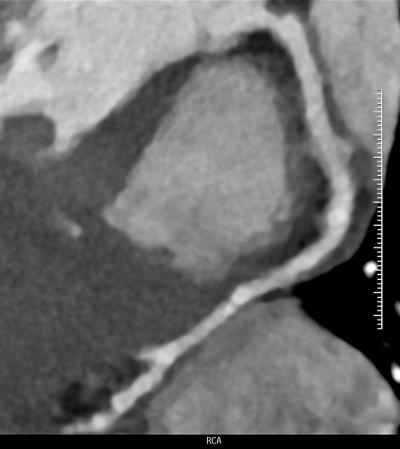

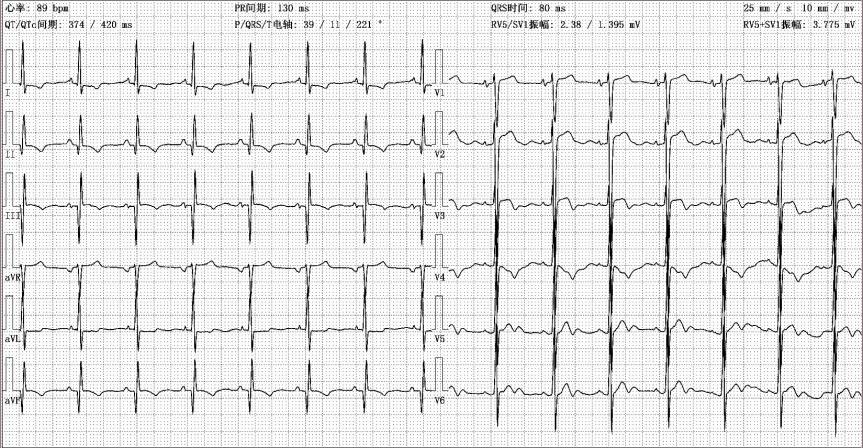

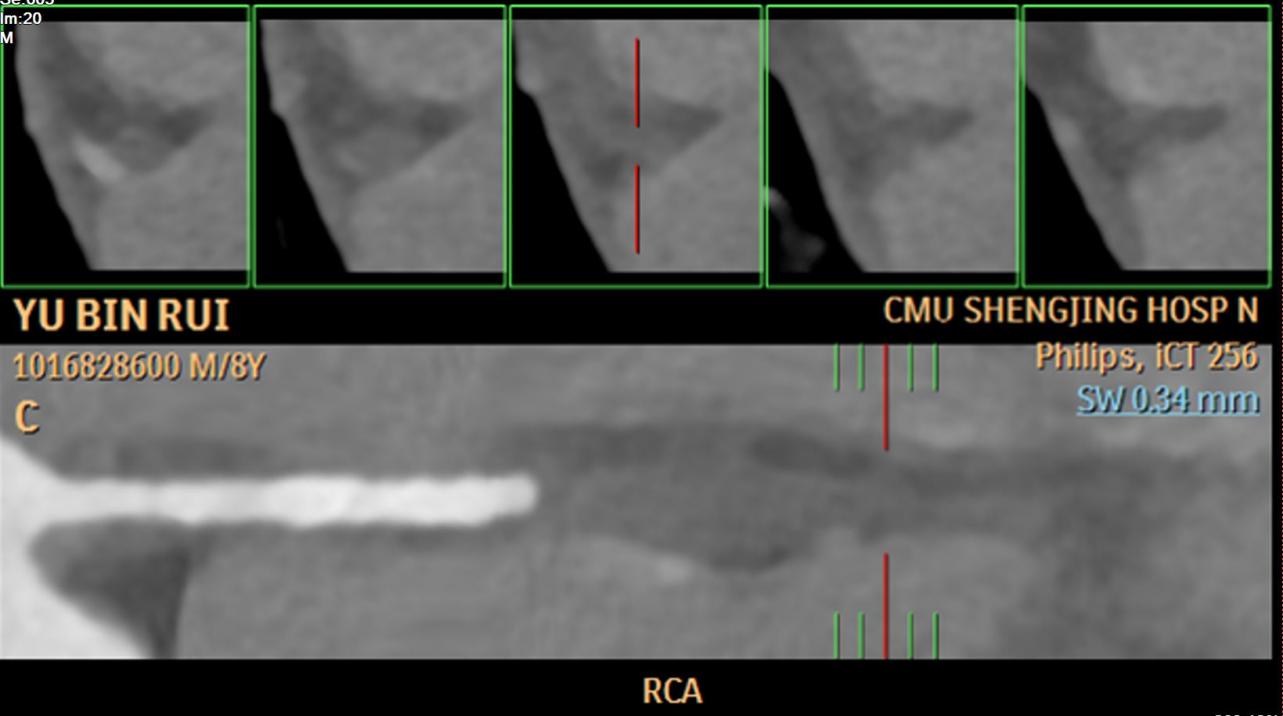


**a**

**e**

**b**

**c**

RCA

thrombus


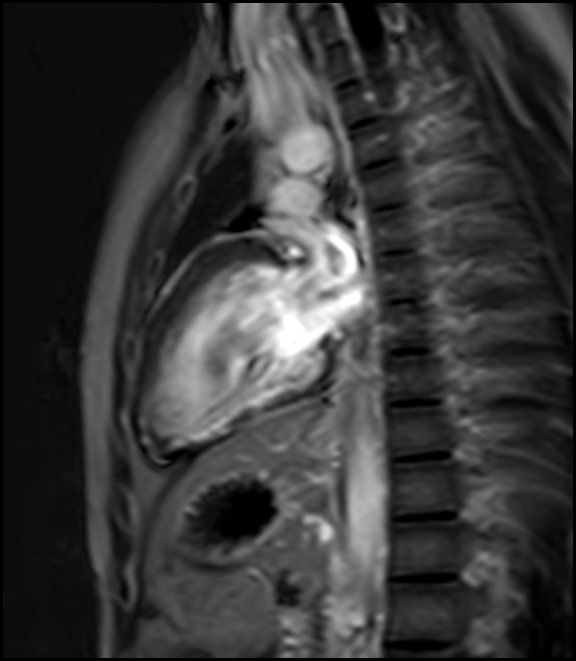


**e**


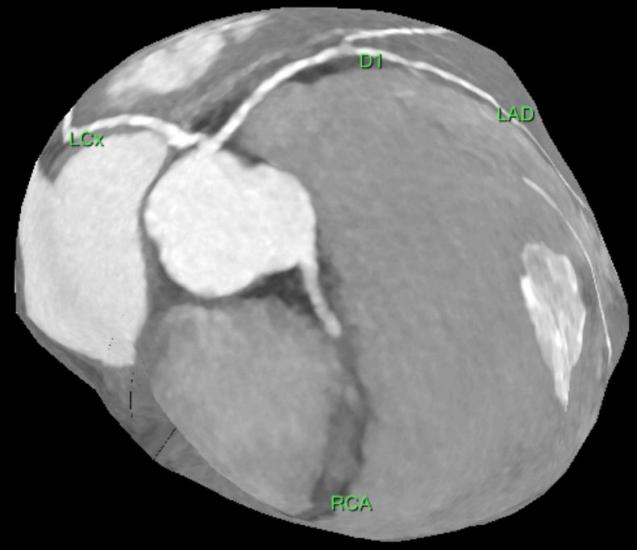


**d**


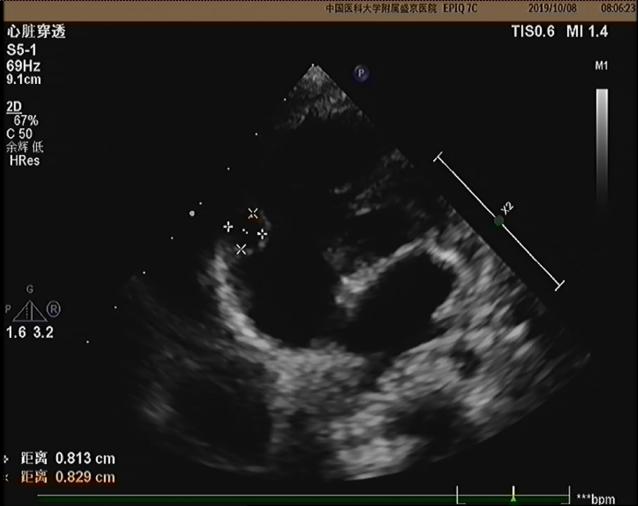

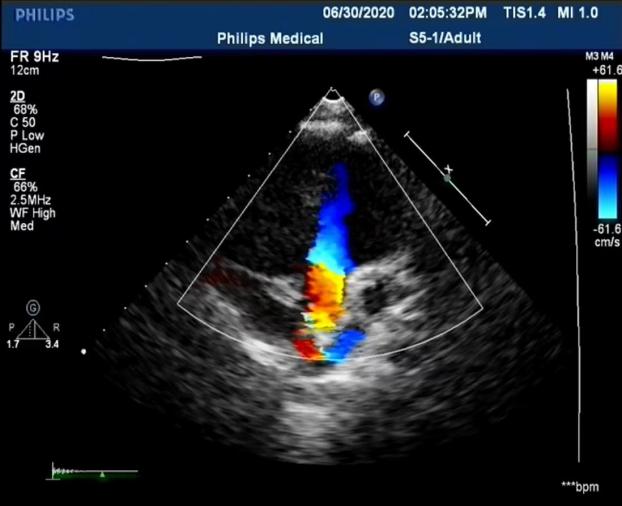

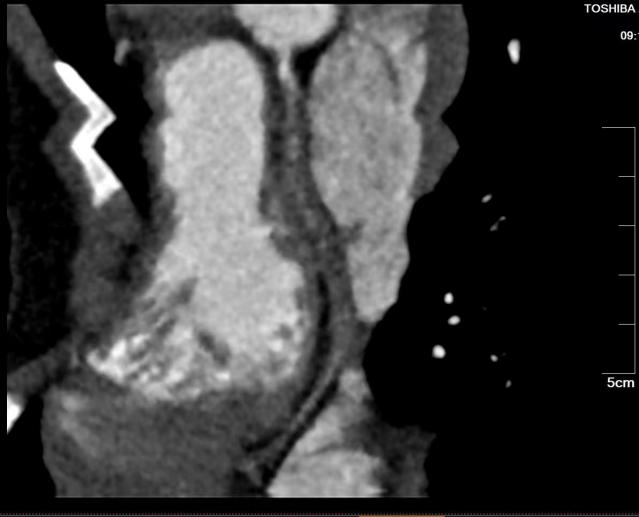

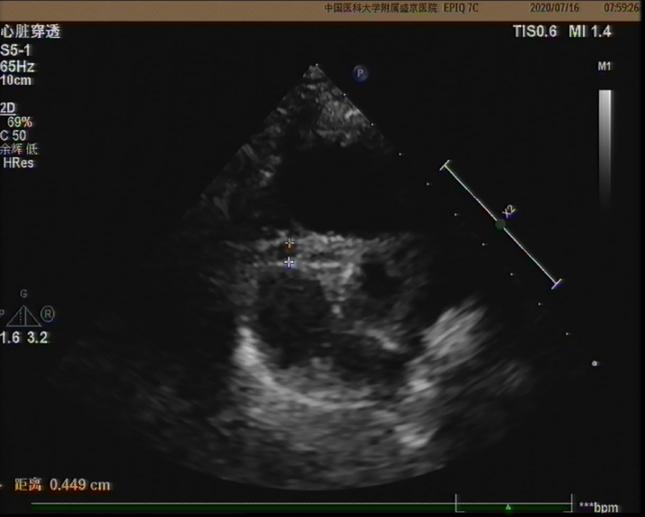


**a**

**d**

**c**

**b**

Figure 9. A boy with KD developed giant RCAA (8.4mm) at the age of 9 months old, 1 month after onset of illness (a). When he was 19 months old, ECHO showed poor color flow filling in the RCA (b), and CTCA showed uneven filling of the proximal middle section of the RCA (c). He was treated with urokinase for 3 days and then heparin for 2 days, subsequent Method 2 for 10 days. The thrombus was successfully dissolved (d).
